# Resistance of Soil-Bound Prions to Rumen Digestion

**DOI:** 10.1371/journal.pone.0044051

**Published:** 2012-08-24

**Authors:** Samuel E. Saunders, Shannon L. Bartelt-Hunt, Jason C. Bartz

**Affiliations:** 1 Department of Civil Engineering, University of Nebraska-Lincoln, Peter Kiewit Institute, Omaha, Nebraska, United States of America; 2 Department of Medical Microbiology and Immunology, Creighton University, Omaha, Nebraska, United States of America; Ohio State University, United States of America

## Abstract

Before prion uptake and infection can occur in the lower gastrointestinal system, ingested prions are subjected to anaerobic digestion in the rumen of cervids and bovids. The susceptibility of soil-bound prions to rumen digestion has not been evaluated previously. In this study, prions from infectious brain homogenates as well as prions bound to a range of soils and soil minerals were subjected to in vitro rumen digestion, and changes in PrP levels were measured via western blot. Binding to clay appeared to protect noninfectious hamster PrP^c^ from complete digestion, while both unbound and soil-bound infectious PrP^Sc^ proved highly resistant to rumen digestion. In addition, no change in intracerebral incubation period was observed following active rumen digestion of unbound hamster HY TME prions and HY TME prions bound to a silty clay loam soil. These results demonstrate that both unbound and soil-bound prions readily survive rumen digestion without a reduction in infectivity, further supporting the potential for soil-mediated transmission of chronic wasting disease (CWD) and scrapie in the environment.

## Introduction

Prion diseases, or transmissible spongiform encephalopathies (TSEs), are fatal neurodegenerative diseases that afflict ruminants, including cattle (bovine spongiform encephalopathy, BSE, or ‘mad cow’ disease), sheep and goats (scrapie), and deer, elk, and moose (chronic wasting disease or CWD), as well as humans (Creutzfeld-Jakob disease or CJD) [1], [2]. The infectious agent of prion diseases is PrP^Sc^, a misfolded isoform of a normal cellular prion protein (PrP^c^) found in all susceptible species [1], [3]. PrP^Sc^ exhibits resistance to proteolysis and inactivation, increased hydrophobicity, and a propensity for aggregation [1], [3]. Moreover, PrP^Sc^ can seed conversion of PrP^c^ to PrP^Sc^ (‘replicate’) and thereby initiate prion propagation and, presumably, disease infection [3].

Natural transmission of CWD and scrapie occurs primarily or exclusively through ingestion or inhalation of prion-contaminated material shed from infected hosts or present in mortalities [2], [4]. Infectious CWD and scrapie prions are shed in saliva, blood, urine, feces, antler velvet, milk, and birthing matter (reviewed by [5]) and are present in the tissue of diseased carcasses [6], [7]. Once ingested by a ruminant (whether sheep, goat, cow, deer, elk, or moose), prions will be subjected to rumen digestion before entering the lower gastrointestinal tract, where agent uptake across the epithelium can initiate infection [8]–[10].

Prions are orally infectious [11], [12] and can be detected in feces following oral inoculation [13], [14] as well as in the feces of diseased animals [15]–[17]. Therefore, it can be assumed that a certain amount of PrP^Sc^ survives the digestive processes in the rumen and lower gastrointestinal system. Results from previous in vitro studies of PrP^Sc^ fate in rumen digestion have been varied. Scherbel and colleagues observed a near-complete loss of 263 K hamster PrP^Sc^, as detected by western blot, following rumen digestion [18]. However, no measurable loss of infectivity was seen in subsequent animal bioassay [19]. Jeffrey et al. observed complete loss of detectable PrP^Sc^ in scrapie-infected sheep brain homogenates following exposure to rumen and other alimentary fluids [9]. However, PrP^Sc^ was detected post-digestion when precipitation and proteinase-K digestion were used prior to western blotting. An additional limited study found no evidence of scrapie PrP^Sc^ digestion in rumen fluids [20]. In sum, these studies demonstrate prions can survive rumen digestion, but it remains unclear whether rumen digestion degrades a significant portion of ingested PrP^Sc^.

Ingestion of prion-contaminated soil has been implicated as a likely mechanism of natural CWD and scrapie transmission [21], but the effect of prion soil sorption on prion susceptibility to rumen digestion remains unknown. Prions bind to a wide range of soils and soil minerals, resist desorption and degradation, remain capable of replication, and retain infectivity [22]–[27]. Alteration of prion infectivity has been observed following soil adsorption [22], [27], but the effect of soil adsorption on prion resistance to degradation remains poorly understood. Effective enzymatic digestion of soil-bound PrP^Sc^ (both CWD-elk and hamster) has been shown previously [23], [24], but this work used a specific subtilisin enzyme known to significantly reduce prion infectivity. Rumen digestion is a complex, highly heterogeneous, anaerobic process carried out by bacteria, protozoa, and fungi primarily targeted at degrading complex carbohydrates and proteins in the ruminant diet [28]. The fate of soil-bound prions may be markedly different in such an environment compared to unbound prions.

Adsorption of prions to soil may alter prion resistance to host degradation, thus potentially altering their oral infectivity and transmissibility. The objective of this research was to evaluate and compare the ability of rumen digestion to degrade unbound prions as well as prions bound to a range of soils and soil minerals. In vitro anaerobic digestion assays seeded by bovine rumen fluid were conducted, and the resultant PrP^Sc^ levels were measured by western blotting. Intracerebral hamster bioassay was also employed to measure changes in infectious titer. The results demonstrate the strong resistance of both unbound and soil-bound prions to rumen digestion, which further supports the efficacy of soil-bound prion ingestion as a natural route of disease transmission in ruminants.

## Methods

### Ethics Statement

All procedures involved in animal bioassay were approved by the Creighton University Institutional Animal Care and Use Committee and complied with the Guide for the Care and Use of Laboratory Animals. Collection procedures for rumen fluid from cannulated dairy cows was approved by the University of Nebraska-Lincoln Institutional Animal Care and Use Committee.

### Prion Source and PMCA Substrate

Prion-infected brain tissue was collected without prior buffer profusion from golden Syrian hamsters infected with the hyper (HY) strain of transmissible mink encephalopathy (TME) as previously described [29]. Uninfected and HY TME brain tissues were homogenized to 10% (w/v) in Dulbecco's phosphate-buffered saline (DPBS) without Ca^++^ or Mg^++^ (Mediatech, Herndon, VA) using strain-dedicated Tenbroeck tissue grinders (Kontes, Vineland, NJ).

### Prion Adsorption

HY TME PrP^Sc^/PrP^c^ and uninfected PrP^c^ from brain homogenates were sorbed to a range of soils as described previously [23]. Gamma-irradiated fine white sand (Fisher Scientific, Pittsburgh, PA), Dickinson sandy loam soil (a Typic Hapludoll), Rinda silty clay loam soil (a Vertic Epiaqualf), sodium bentonite clay (CETCO, Arlington Heights, IL), and silicon dioxide powder (SiO_2_, Sigma Aldrich, St. Louis, MO) were used and have been described previously [23], [26]. Briefly, to obtain soil-bound PrP, 10% brain homogenate was combined with soil in 1X DPBS and gently rotated at 22°C, then centrifuged at 100 g for 5 min. The supernatant was removed, and the pellets were washed 2 times with DPBS. PrP adsorption to silty clay loam, bentonite clay, and SiO2 powder adsorption was conducted in 15 ml polypropylene tubes (Fisher Scientific). PrP adsorption to sandy loam and quartz sand was conducted in 0.2 ml polypropylene PCR tubes (Fisher Scientific). The final pellets were collected and stored at −80°C. Incubation times, as well as soil, buffer, and homogenate∶soil ratios were as reported previously [27] (Table S1) and selected to achieve maximum or near-maximum PrP adsorption based on previous results [25], [26].

### Rumen Digestion Assay

Standard in vitro rumen digestion assay methods were followed [18], [30], [31]. Active rumen matter was collected from healthy cannulated dairy cows on a single farm approximately 5 hours after feeding. Standard dairy cow diets were used, consisting of corn silage, sweet bran feed, and brome and alfalfa hays, but diet was not constant for all samplings and multiple cows were used over the course of the study. Percent grain ranged from 23–60%. No difference in immunoblot results was observed across all diets used (data not shown), although an extensive study of this variable was not conducted. Immediately after collection, rumen matter was hand-pressed through two layers of cheesecloth to remove large feed particles and sealed in a warmed thermos bottle with minimal headspace. The fluid was transported (45 min) to the lab and placed in an anaerobic chamber with an atmosphere of 85% N_2_, 10% H_2_, and 5% CO_2_. Rumen fluid was diluted 1∶10 or 1∶5 in McDougall's buffer (simulating ruminant saliva, 10.5 mM KCl, 8 mM NaCl, 0.5 mM MgSO_4_, 0.4 mM CaCl_2_, 0.11 M NaHCO_3_, 27 mM Na_2_HPO_4_, pH 8.3) with soluble carbohydrates (6.7 g/L maltose, 3.3 g/L xylose, 3.3 g/L soluble starch, 2.1 g/L NaHCO_3_, 3.3 g/l citrus pectin). There was no difference in immunoblot results between 1∶10 and 1∶5 rumen∶buffer dilutions (data not shown).

Resazurin dye (Acros Organic, New Jersey) was used as an indicator of redox state and does not affect in vitro digestion [32]. For all active digestions, resazurin dye added to active rumen solutions remained colorless throughout the incubation, indicating highly-reduced, anaerobic conditions prevailed. For inactive controls, rumen fluid was autoclaved at 121°C for 15 min. Active or inactive rumen fluid or buffer (McDougall's with soluble carbohydrates) was combined with prion-infected brain or soil homogenates at a ratio of 5∶1 (rumen buffer∶prion homogenates) in 0.2 ml PCR tubes. Rumen-prion mixtures were vortexed and then incubated at 39°C for 20 hr with occasional (≈6 hr) cap venting. Following incubation, samples were stored at −80°C until analyzed. The average pH of the in vitro digestions is shown in Table 1.

**Table 1:** pH of in vitro rumen digestions.

Sample	Contents	Incubation^1^	Average pH^2^
Buffer	Buffer, Carbohydrates, Brain Homogenate	0	8.6±0.1
		20	8.2±0.1
Inactive digestion	Buffer, Carbohydrates, Brain Homogenate, Inactive Rumen Fluid	0	8.6±0.6
		20	8.3±0.6
Active digestion	Buffer, Carbohydrates, Brain Homogenate, Active Rumen Fluid	0	7.6±0.4
		20	6.2±0.2

1 hr at 39°C.

2 n = 3–4, mean ± Std. dev.

### Immunoblot Analysis

**Figure 1:**
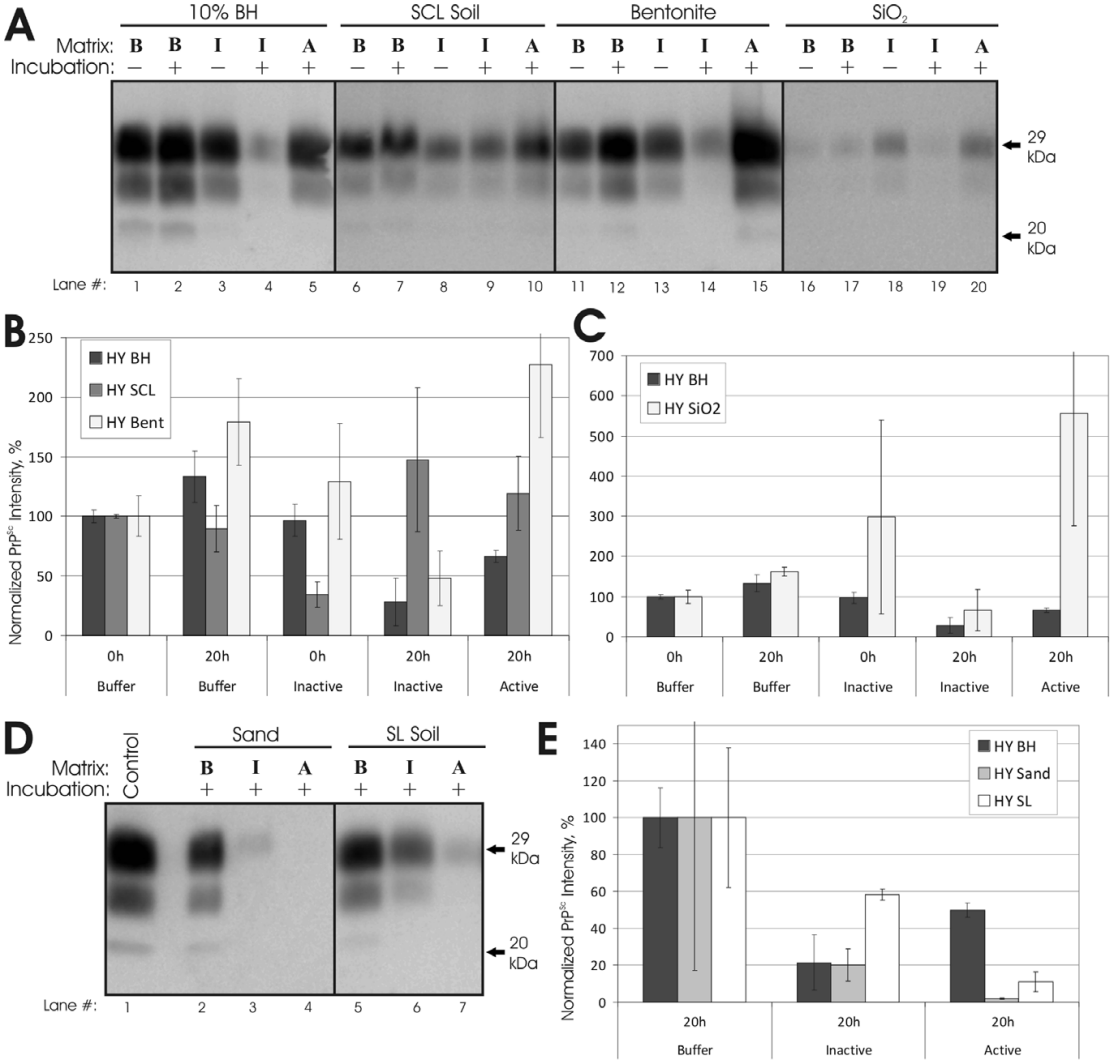
Rumen digestion of unbound and soil-bound HY TME PrP^Sc^. (**A and D**): Representative immunoblots (n = 3–6) of HY TME in vitro rumen digestions. ‘B’: samples in McDougall's buffer with carbohydrates. ‘I’ samples in inactivated rumen buffer. ‘A’: samples in active rumen buffer. Incubation length was 20 hr at 39°C. All samples treated with proteinase K. (**B, C, and E**): Quantification of immunoblots. (B): Unbound (brain homogenate, BH), silty clay loam (SCL) Soil, and bentonite clay. (C): SiO2 powder. (E): Sand and sandy loam (SL) soil. All samples were normalized to the buffer 0 hr samples, except sand and SL soil samples (E) were normalized to the buffer 20 hr samples. Error bars show ±1 standard error of the mean.

Detection of PrP^Sc^ in digested and undigested samples was accomplished using SDS-PAGE/Western blotting as described previously without modification [23], [33]. Briefly, for proteinase K (PK) treatment, sample aliquots were incubated at 37°C under constant agitation for 1 hr with 25 µg PK per ml of sample (Roche Diagnostics Corporation, Indianapolis, IN). PK digestion was stopped by boiling in SDS-PAGE sample buffer. Soil sample amounts loaded into gels are reported in (Table S1). Samples were separated on 12.5% acrylamide gels under reducing conditions and transferred to polyvinyl difluoride (PVDF) membranes. All hamster samples were immunoblotted with mAb 3F4 (Millipore, Billerica, MA, 1∶10,000 dilution). Blots were developed with Pierce Supersignal West Femto maximum-sensitivity substrate and imaged on a Kodak 2000R imaging station (Kodak, Rochester, NY). None of the soils used exhibit nonspecific binding to either the primary or secondary antibody [23]. Rumen content also did not exhibit nonspecific binding (see, e.g., Figure 1D, lane 4). Blot images were quantified using Kodak 1D 4.0 software (Kodak, Rochester, NY), which output the net intensity of each blot (total darkness minus background). Net intensities of sample replicates (n = 3 to 6) were normalized as a percentage of the average of control HY BH replicates (n = 4) run on the same gel to control for inter-gel variance.

### Animal Bioassay

Intracerebral inoculations of male golden Syrian hamsters (Harlan Sprauge-Dawley, Indianapolis, IN) were conducted as described previously [34] with five animals per group. Samples of rumen-digested and undigested HY TME bound to silty clay loam soil or unbound were gamma irradiated (8 kGy) and diluted 1∶10 in DPBS and then 25 µl was inoculated per animal. The incubation period was calculated as the length of time in days between inoculation and the onset of clinical signs that include ataxia and hyperactivity to external stimuli.

### Statistical Analysis

Two-tailed student's T-tests assuming unequal variances were performed using Microsoft Excel to determine statistical significance as noted. P values less than 0.05 were considered statistically significant.

## Results and Discussion

### Resistance of Unbound PrP^Sc^ to Rumen Digestion

An in vitro digestion assay was employed to simulate rumen digestion of prion-contaminated material. Standard methods, including standard rumen fluid sampling procedures and substrate and buffer compositions, were used [30], [31], [35]. pH values for the in vitro digestion were within normal in vivo ranges (Table 1), resazurin dye indicated a reduced environment in active samples but not inactive and buffer controls, and gas was produced throughout the 20 hr incubation, indicating anaerobic digestion occurred.

Unbound HY TME PrP^Sc^ from brain homogenate was not significantly reduced in actively digested samples compared to inactive and buffer controls (Figures 1A, lanes 1–5 and 1B). Incubation up to 48 hr did not yield significant degradation (data not shown). Immunoblots of actively digested samples not subjected to proteinase-K exhibited a shift in migration (Figure S1), indicative of PrP^Sc^ N-terminal truncation and suggest limited proteolysis of PrP^Sc^ did occur [33]. However, the PrP^Sc^ N-terminus is not required for prion infectivity [36]. Preliminary results with hamster DY TME, elk CWD, sheep scrapie, bovine TME, and mink TME also showed no differences in unbound PrP^Sc^ in digested samples and controls (data not shown).

These results are consistent with the result of Nicholson and colleagues [20] showing no decrease in scrapie PrP^Sc^ following in vitro rumen digestion, but somewhat inconsistent with Scherbel et al. [18], who observed significant (near-complete) 263 K hamster PrP^Sc^ degradation during active digestion in the absence of detergents. Methodological differences such as rumen fluid seed or western blotting techniques may be responsible for the observed differences. For instance, we collected rumen fluid from a live, cannulated dairy cow while Scherbel et al. collected fluid from a slaughtered beef bull.

### Resistance of Soil-Bound PrP^Sc^ to Rumen Digestion

To determine the effect of soil on the susceptibility of prions to rumen digestion, PrP^Sc^ was sorbed to a range of soils and soil minerals and exposed to in vitro rumen digestion. Consistent with the unbound results, HY PrP^Sc^ bound to silty clay loam (SCL) soil was not reduced following active rumen digestion (Figures 1A, lanes 6–10, and 1B). Preliminary results for CWD-elk PrP^Sc^ bound to SCL soil demonstrated similar resistance to digestion (data not shown). Increased detection of PrP^Sc^ bound to bentonite clay (Figures 1A, lanes 11–15, and 1B) and silicon dioxide powder (SiO_2_) (Figures 1A, lanes 16–20, and 1C) was observed in digested samples compared to controls. These bentonite and SiO_2_ results were highly variable, especially SiO_2_, but suggest that active rumen digestion increased PrP^Sc^ desorption and detectability. PrP^Sc^ detection from SiO_2_ in all samples, including buffer controls, was very low (1–4% recovery, Figure 1A, lanes 16–20). This contrasts with previous results reporting SiO_2_ PrP^Sc^ recoveries equal to or greater than 100% in three other aqueous solutions [37]. Because PrP^Sc^ recovery from other soils and unbound samples in buffer was not abnormal (Figure 1A), the low PrP^Sc^ recoveries from SiO_2_ may be due to a specific chemical effect on the mineral particles (that in turn alters PrP^Sc^ desorption) and not a direct effect on PrP^Sc^.

HY TME bound to sandy loam (SL) soil and sand was susceptible to rumen digestion (Figures 1D and 1E), and PrP^Sc^ was not detected on sand samples actively digested (Figure 1D, lane 4). However, the SL soil and sand results were highly variable and not statistically-significant from undigested controls. Further study may yield more precise data on PrP^Sc^ resistance to digestion when bound to these soils, but preliminary PMCA data indicates sand-bound PrP^Sc^ remains capable of replicating following active digestion (discussed below).

### Rumen Digestion of PrP^c^

**Figure 2:**
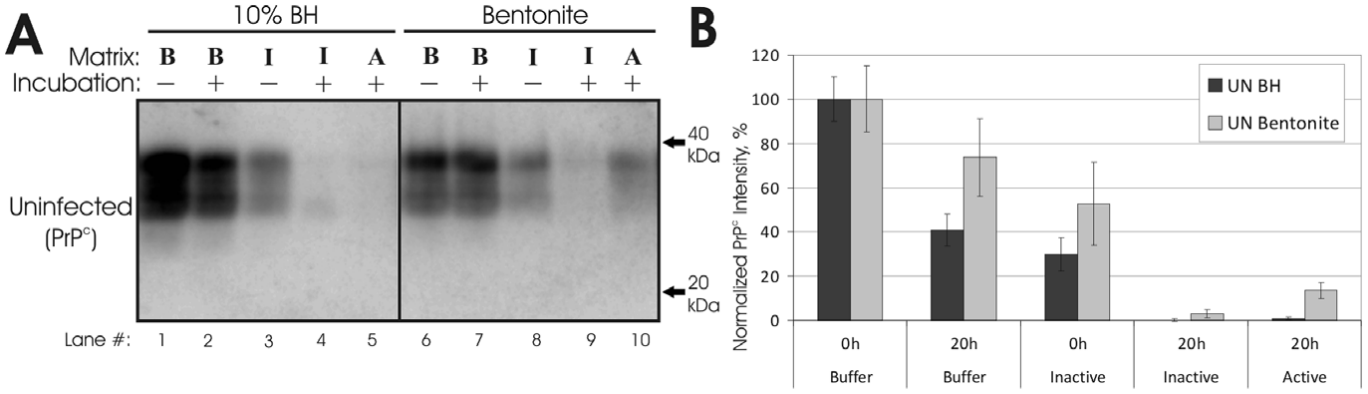
Rumen digestion of bound and unbound PrP^c^. (**A**): Representative immunoblots (n = 3–6) of hamster PrP^c^ in vitro rumen digestion. ‘B’: samples in McDougall's buffer with carbohydrates. ‘I’ samples in inactivated rumen buffer. ‘A’: samples in active rumen buffer. No samples were treated with proteinase K. (**B**): Quantification of immunoblots. Samples were normalized to the buffer 0 hr samples. Error bars show ±1 standard error of the mean.

Rumen digestion was completely effective at degrading PrP^c^ from uninfected hamster brain homogenate (Figure 2A, lane 5, and 2B), consistent with previous studies [18], [20]. This result typifies the increased resistance to proteolysis of PrP^Sc^ compared to PrP^c^ [23] and illuminates a practical effect of this increase on disease transmission: PrP^Sc^ is able to survive rumen digestion whereas PrP^c^ is not. A 60% decrease in detectable PrP^c^ was observed for samples incubated in buffer (Figure 2A, lane 2), and only a faint PrP^c^ signal was detected in samples incubated in inactive rumen content (Figure 2A, lanes 4). Thus, noncellular physical or chemical mechanisms were most likely responsible for the decreases in PrP^c^ observed in the actively digested samples. These mechanisms could include irreversible sorption to rumen particles, heat degradation, or enzymatic degradation (from enzymes introduced in the brain homogenate [33]).

In contrast to unbound PrP^c^, PrP^c^ bound to bentonite clay was still detected following inactive and active digestion (Figure 2A, lane 9). This suggests that PrP^c^ sorption to bentonite may increase its resistance to rumen degradation, perhaps due to a decrease in access to cleavage sites. As with unbound PrP^c^ samples, PrP^c^ levels in inactive bentonite controls were reduced (Figure 2A, lane 8), again implicating physiochemical mechanisms of PrP^c^ degradation.

### Infectivity of Unbound and Soil-Bound Prions Following Rumen Digestion Is Unchanged

Hamsters were inoculated with unbound and SCL-bound HY TME prions subjected to either active or inactive (pre-autoclaved) in vitro rumen digestion. The incubation periods of hamsters inoculated with inactive or active samples were equal (Table 2). The incubation periods for the inoculated dose were consistent with previous studies [27], [34]. All animals inoculated exhibited classic HY TME clinical symptoms, and all clinical animals contained HY PrP^Sc^ in the central nervous system (CNS) (data not shown). Since there is a well-established relationship between HY TME infectious titer and incubation period [34], including for soil-bound HY TME [27], these results strongly suggest rumen digestion does not alter HY TME infectious titer for either unbound or soil-bound prions. These data are consistent with the results of Scherbel and colleagues, who also observed no difference in attack rate or incubation period between (unbound) 263 K-strain hamster prions subjected or not subjected to in vitro rumen digestion [19]. Furthermore, these data also correlate with our western blot results, which demonstrated no difference in PrP^Sc^ levels before and after digestion in unbound and SCL soil-bound PrP^Sc^ (Figures 1A and 1B).

**Table 2:** Rumen digestion does not alter the incubation period of HY TME.

Sample	Digestion Treatment	Mean Incubation Period^1^
HY unbound	Inactive	71 (±3)
HY unbound	Active	71 (±3)
HY SCL soil-bound	Inactive	84 (±3)
HY SCL soil-bound	Active	84 (±3)

1 Days (± standard error of the mean). Attack rate (# inoculated/# diseased) was 5/5 for all groups.

Also consistent with previous results, the mean incubation time of SCL soil-bound HY TME was significantly longer (13 d) than unbound HY TME (Table 2) [27]. This increase in incubation period correlates with a 1.2-log decrease in infectious titer of HY TME upon binding to SCL soil and a similar decrease in HY TME PrP^Sc^ replication efficiency [27]. Thus, the present results indicate SCL-bound prions remain less infectious than unbound prions following rumen digestion.

### Implications for Environmental Prion Transmission

To initiate infection via absorption across the lower gastrointestinal epithelium, orally ingested prions must survive passage through the rumen [8]–[10]. Previous studies have observed varied PrP^Sc^ resistance to in vitro rumen digestion [9], [18], [20]. We observed that active in vitro rumen digestion did not reduce PrP^Sc^ abundance (Figure 1), and consistent with the previous work of Scherbel et al. [19], unbound prion infectivity was not reduced following rumen digestion (Table 2). Moreover, our results demonstrate that PrP^Sc^ sorption to soil does not reduce prion resistance to rumen digestion. However, since both unbound and soil-bound prions were resistant to rumen digestion, we cannot conclude that soil sorption increases prion resistance to gut degradation, only that it does not decrease it. Nevertheless, the resistance of soil-bound prions to rumen digestion supports the efficacy of soil-mediated prion transmission (prion-soil sorption and subsequent ingestion or inhalation by a naïve host) [21] as a natural mechanism of CWD and scrapie transmission.

We did observe variance in PrP^Sc^ resistance to digestion with respect to soil type, where, in contrast to the other soils and minerals, PrP^Sc^ levels bound to sand and sandy loam soil were reduced following digestion (Figure 1D and 1E). Variance in prion-soil interactions of this kind could lead to spatial variance in prion disease incidence based on local soil-type [21]. However, preliminary protein misfolding cyclic amplification (PMCA) experiments [27] indicate the replication efficiency of prions subjected to active digestion while bound to sand or SiO_2_ is not significantly different than unbound prions (data not shown). Based on the established relationship between PMCA replication efficiency and infectious titer [24], [27], these results suggest the SCL soil bioassay results are typical of the other soils and soil minerals used. Still, bioassay of other soils is needed to definitively evaluate soil-type variance in digestion resistance.

A number of factors must be considered in extending the present results. First, the results were obtained using in vitro digestion, which is a simulation of in vivo processes with limitations [30], [35]. We used standard in vitro methods, consistent with previous prion digestion studies, although the limited amount of prion-infected brain homogenate available necessitated using small (0.2 ml) tubes, which may have contributed to the observed variance. Second, prion resistance to digestion may vary with prion strain and species [23], [33]. As noted above, our preliminary work with other prion strains and species suggests broad prion resistance to rumen digestion, but these results would need to be confirmed with additional studies. Third, rumen digestion can vary with host species and diet, with the latter appearing more significant than the former [38]. Studies have reported similar in vitro digestion (as measured by parameters such as gas production and substrate utilization) when using rumen fluid contents from sheep and cows [39], [40], sheep and goats [41], and sheep and red deer [42] when animals were fed the same diet. Variance in the diet of the cows used to collect rumen fluid (23–66% grain) did not observably affect the immunoblot results of this study (data not shown), suggesting that diet is not a significant factor and that our results are applicable across a wide range of diets and species (cervids, sheep, goats, and cattle). However, an extensive study of the effect of diet was not conducted. Moreover, dairy cow diets are notably different than free-ranging deer diets, and deer diets vary seasonally as well as geographically, which can affect rumen digestion [43].

Finally, unbound and soil-bound prions surviving rumen passage will be exposed to stomach and intestinal digestion before uptake. These two processes are less complex than rumen digestion, and previous results indicate unbound prions are resistant to both [8], [9], [44]. Still, the effect of soil sorption on prion resistance to lower gastrointestinal digestion has yet to be investigated. Moreover, while passage through the rumen and lower gastrointestinal tract may not digest PrP^Sc^, it may alter PrP^Sc^ uptake efficiency, which would not be detected by immunoblot or intracerebral bioassay. Thus, study of soil-bound prions in, for example, the gut-loop system employed by Dagleish and Jeffery [8], [9] would be of interest in further evaluating the efficacy of soil-mediated prion transmission.

## Supporting Information

Figure S1 **Rumen digestion of unbound HYTME PrP^Sc^ without proteinase-K treatment.**
(DOCX)

Table S1 **PrP Adsorption to Soil and Soil Minerals.**
(DOCX)
