# Supplementary material for: Resistance of Soil-Bound Prions to Rumen Digestion
Source: PLoS One. 2012 Aug 24;7(8):e44051. doi: 10.1371/journal.pone.0044051 (PMC3427226; doi:10.1371/journal.pone.0044051)
Supplement: Figure S1 — Rumen digestion of unbound HYTME PrPSc without proteinase-K treatment. (DOCX) [file pone.0044051.s001.docx]

**Resistance of Soil-Bound Prions to Rumen Digestion**

SAMUEL E. SAUNDERS^1^, SHANNON L. BARTELT-HUNT^1^, AND JASON C. BARTZ^2^

^1^*Department of Civil Engineering, University of Nebraska-Lincoln, Peter Kiewit Institute, Omaha, Nebraska, United States of America,* ^2^*Department of Medical Microbiology and Immunology, Creighton University, Omaha, Nebraska, United States of America*


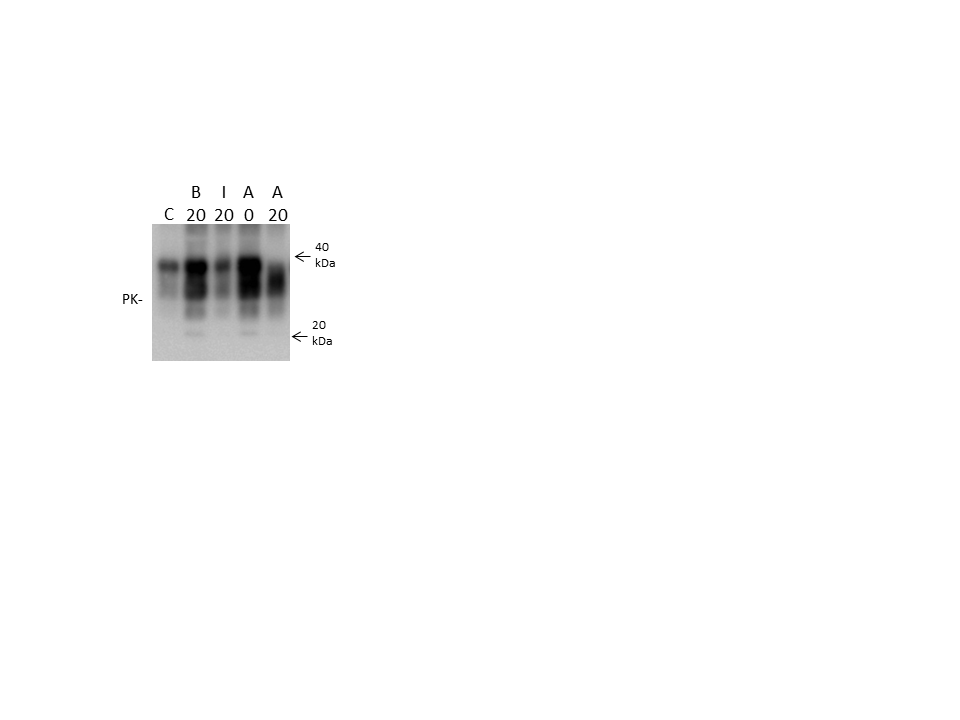


**Figure S1.** Rumen digestion of unbound HYTME PrP^Sc^ without proteinase-K treatment. Representative immunoblots of hamster PrP^Sc^ in vitro rumen digestion. ‘B’: samples in McDougall’s buffer with carbohydrates. ‘I’ samples in inactivated rumen buffer. ‘A’: samples in active rumen buffer. ‘20’: samples incubated for 20 hr at 39°C.
