# Supplementary material for: Resistance of Soil-Bound Prions to Rumen Digestion
Source: PLoS One. 2012 Aug 24;7(8):e44051. doi: 10.1371/journal.pone.0044051 (PMC3427226; doi:10.1371/journal.pone.0044051)
Supplement: Table S1 — PrP Adsorption to Soil and Soil Minerals. (DOCX) [file pone.0044051.s002.docx]

**Resistance of Soil-Bound Prions to Rumen Digestion**

SAMUEL E. SAUNDERS^1^, SHANNON L. BARTELT-HUNT^1^, AND JASON C. BARTZ^2^

^1^*Department of Civil Engineering, University of Nebraska-Lincoln, Peter Kiewit Institute, Omaha, Nebraska, United States of America,* ^2^*Department of Medical Microbiology and Immunology, Creighton University, Omaha, Nebraska, United States of America*

**Supplemental Materials:**

**Table S1.** PrP Adsorption to Soil and Soil Minerals

| **Soil/**  **Mineral** | **Adsorption Incubation Time** | **Soil Amount** | **DPBS**  **Buffer Amount** | **10% Brain Homogenate** | **Amount**  **Imaged** |
| --- | --- | --- | --- | --- | --- |
|  | hr | mg | ml | µl | mg |
| Rinda Silty Clay Loam | 24 | 50 | 10 | 500 | 0.25 |
| Bentonite Clay | 24 | 25 | 10 | 500 | 0.25 |
| SiO_2_ Powder | 24 | 100 | 10 | 500 | 2.5 |
| Dickinson Sandy Loam | 168 | 3 | 0.2 | 10 | 3 |
| Fine Quartz Sand | 168 | 10 | 0.2 | 10 | 10 |
